# Design and characterisation of a novel interleukin-15 receptor alpha fusion protein and analysis of interleukin-15 complexation

**DOI:** 10.1371/journal.pone.0219313

**Published:** 2019-07-26

**Authors:** Anja Sophie Schmid, Dario Neri

**Affiliations:** Department of Chemistry and Applied Biosciences, Swiss Federal Institute of Technology (ETH Zürich), Vladimir-Prelog-Weg, Zürich, Switzerland; King's College London, UNITED KINGDOM

## Abstract

Interleukin-15 (IL15) is one of the most important cytokines currently being considered for cancer therapy applications. It is thought that by administering IL15 in complex with its cognate receptor alpha chain (IL15Rα) its biological activity could be increased manifold. We produced a fusion protein of mouse IL15Rα and the F8 antibody, that targets the alternatively-spliced extra-domain A (EDA) of fibronectin, which is overexpressed in many types of cancer. The fusion protein F8IL15Rα was cloned, expressed and characterized *in vitro* and its ability to bind to mouse IL15 was assessed with both size exclusion chromatography (SEC) and surface plasmon resonance (SPR) experiments. Furthermore, mouse and human IL15 and their corresponding Fc fused IL15Rα subunits were purchased, characterized and used to compare the capacity of F8IL15Rα to generate complexes. Surprisingly, none of the IL15Rα fusion proteins showed IL15 complexation on SEC. However, on SPR, F8IL15Rα displayed the ability to bind IL15. In a cell-based activity assay none of the IL15Rα fusions were able to increase cellular proliferation in combination with IL15 compared to IL15 alone. A better understanding of the molecular requirements for effective IL15 signalling are likely to be important for the development of IL15-based biopharmaceuticals.

## 1 Introduction

Interleukin-15 (IL15) is a cytokine that has been hailed as one of the most promising agents in the fight against cancer.[1, 2] It is produced by dendritic cells, monocytes, fibroblasts and epithelial cells and has similar roles to those of interleukin-2 (IL2), being involved in T cell, B cell and natural killer (NK) cell proliferation.[3, 4] However, while IL2 supports regulatory T cell (Treg) maintenance and activation-induced cell death (AICD), leading to apoptosis of stimulated T cells as well as induction of T cell tolerance, IL15 does not seem to have a major effect on Tregs and actually inhibits AICD.[5] Furthermore, not only is IL15 involved in the generation of long-lasting CD8+ memory T cells, but studies in animals have also shown a more desirable safety profile for IL15 compared to IL2, as no capillary leak syndrome was observed.[6, 7] Taking all of this into account, IL15 is thought to be an even more suitable candidate than IL2 to achieve cancer cures. Important caveats in the therapeutic usage of IL15, however, are the low production yields and numerous difficulties of protein production, especially using mammalian cells.[7]

An intriguing aspect of IL15 biology is its trimeric IL15 receptor (IL15R), which is very similar to that of IL2. The receptors share the same two signalling subunits, namely IL2 receptor beta (IL2Rβ) and the common gamma chain (γ_c_), whilst possessing individual alpha receptor chains (IL15Rα and IL2Rα, respectively), which increase receptor affinity.[2] It is widely debated as to how the two cytokines can possess different biological functions although they signal through the same receptor subunits, but it is thought to involve either conformational aspects, spatial and temporal receptor subunit distribution, the recruitment of different signalling mediators or differences in cis- and trans-presentation by the alpha subunits.[2] IL15Rα, consisting of a sushi-domain (responsible for cytokine binding), a linker, a proline-threonine rich and a short cytoplasmic region, mainly presents IL15 in trans and has an extremely high affinity to the cytokine (approx. 30–100 pM).[4, 8] Multiple studies have shown, that the biological effects of IL15 can be enhanced by complexing and delivering it with IL15Rα.[3, 5, 9–12] This has led to the development of a number of IL15-based therapeutics, including ALT-803, a complex between a mutated IL15 superagonist and an IL15Rα sushi-domain crystallisable fragment (Fc) fusion, which is currently being investigated in a phase II clinical trial.[5, 13]

Our laboratory has described numerous fusion proteins of disease-targeting antibodies and antibody fragments coupled to, amongst others, a variety of cytokine payloads, including those featuring IL15 as bioactive moiety. [14–16] IL15-based fusion proteins capable of inhibiting tumour growth were generated, but those products were not able to efficiently localize to tumours at low doses, as a result of an *in vivo* trapping mechanism.[16, 17] When considering antibodies for tumour targeting applications, the F8 antibody is one of the most promising candidates. This antibody specifically recognizes the alternatively-spliced extra-domain A (EDA) of fibronectin, which is expressed at high levels both in inflammatory diseases as well as in numerous cancers.[18, 19] EDA is virtually absent from normal tissues (with the exception of the female reproductive tract), while strongly overexpressed at sites of new blood vessel formation and tissue remodelling.[19, 20]

The goal of this study was to design and produce a novel fusion protein (F8IL15Rα), consisting of the F8 antibody in diabody format fused to the extracellular portion of mouse IL15Rα, with the plan to non-covalently assemble the product with recombinant IL15 and use the resulting complex for tumour targeting applications. In order to verify the complex formation process, mouse and human IL15Rα-Fc fusions were purchased and the capabilities of the three fusion proteins to form complexes with IL15 and increase IL15-dependent cellular proliferation were assessed and compared. Although F8IL15Rα was able to bind IL15 in surface plasmon resonance experiments, no complexation was observed using size exclusion chromatography and no increased activity in a cellular activity assay was reported for any of the produced or bought IL15Rα-fusions with IL15 compared to IL15 alone.

## 2 Material and methods

### 2.1 Cell culture

CHO-S cells (Invitrogen, Switzerland) were cultured in suspension either in shaking incubators (37°C, 160 rpm) in PowerCHO-2 CD (LZ-BE12-771Q; Lonza, Belgium) or for protein production in shaking incubators (31°C, 135 rpm) in ProCHO 4 (LZ-BE12-029Q; Lonza, Belgium), both mediums supplemented with 1X HT Supplement (50X) (Gibco, UK), 4 mM Ultraglutamine 1 (Lonza, Belgium) and 1X Anti-Anti (100X) (Gibco, UK).

CTLL2 cells were cultured in RPMI 1640 (Gibco, UK) supplemented with 2 mM Ultraglutamine 1 (Lonza, Belgium), 25 mM Hepes (Gibco, UK), 10% Fetal Bovine Serum (Gibco, UK), 0.05 mM β-Mercaptoethanol, 1X Anti-Anti (100X) (Gibco, UK), and 60 Units/mL Interleukin-2, human (Roche Diagnostics GmbH, Germany).

### 2.2 Cloning of F8IL15Rα

The F8 antibody in diabody (Db) format has been described previously.[18, 21] The F8 antibody in diabody format (containing an N-terminal secretion signal and NheI restriction site) and linked C-terminally with a 15-amino-acid linker (SSSSG)_3_ to the extracellular portion of mouse IL15Rα_(33–205),_ which in turn was linked C-terminally to mouse IL15_,_ was ordered from Genscript (United States) in the mammalian expression vector pcDNA3.1(+).The desired construct was generated with polymerase chain reaction (PCR) by amplifying the F8IL15Rα region with the primer pair 5’ CCCGCTAGCGTCGACCATGGGCTGGAGCCTGATCCTCCTGTTCCTCGTCGCTGTGGC 3’ and 5’ CCCCGCGAATTCTCATTAAGCTATTTCGTCATTTTGGAACTGTGGGGAGAAATCTCTG 3’, adding a C-terminal stop codon and restriction site EcoRI. F8IL15Rα was digested with NheI and EcoRI and ligated into the mammalian expression vector pcDNA3.1(+) (Invitrogen).

### 2.3 Transient gene expression, protein purification and protein characterisation

F8IL15Rα was generated by polyethyleneimine (PEI)-mediated transient gene expression in CHO-S cells.[22] For a 1 mL production, 10^6^ CHO-S cells were centrifuged, resuspended in 0.5 mL ProCHO 4 medium and 0.9 μg of F8IL15Rα plasmid DNA and 2.5 μg of PEI (Polysciences) were added. The cell suspension was then incubated at 31°C for 6 days in a shaking incubator. To obtain a desired production volume of 800 mL, the procedure was scaled up. Protein A affinity chromatography was used for protein purification from the culture supernatant.

All proteins were analysed using size exclusion chromatography (with a flow of 0.75 mL/min in PBS) (Superdex 200 increase, 10/300 GL) (GE Healthcare, UK) and SDS-PAGE in non-reducing and reducing conditions.

### 2.4 Other protein reagents

Recombinant mouse IL15 (named muIL15 peprotech) (PeproTech, #210–15) and (named muIL15 biolegend and muIL15 biolegend batch 2) (Biolegend, 566304), recombinant human IL15 (named huIL15) (PeproTech, #200–15), a recombinant mouse IL15Rα_(33–205)_-Fc chimera (named muIL15RαFc) (R&D Systems, 551-MR-100), a recombinant human IL15Rα_(1–205)_-Fc chimera (named huIL15RαFc) (R&D Systems, 7194-IR-050) and an anti-mouse IL15 monoclonal antibody (named aIL15mAb) (ThermoFisher 201136, MA5-23786) were purchased.

### 2.5 Complexation experiments

Complexes of IL15Rα fusion proteins and IL15 were formed by mixing the respective proteins suspended in PBS and incubating them at 37°C for 20 minutes.[9, 11] The same conditions were used for the positive control complexation of F8 IL15Rα with EDA. Alternative conditions in supplementary material are indicated. Molar concentration ratios of IL15Rα fusion protein monomers to IL15 were varied and are indicated in the respective figures. All complexes were analysed with size exclusion chromatography (Superdex 200 increase, 10/300 GL).

The ability of F8IL15Rα to bind mouse IL15 was furthermore analysed with surface plasmon resonance analysis (BIAcore S200) (GE Healthcare, UK) on an EDA antigen-coated CM5 Biacore sensor chip. 500 nM F8IL15Rα was injected followed by IL15 samples at indicated concentrations at a flow of 20 μL/min, using both phosphate buffered saline (PBS) and 500 nM bovine serum albumin (BSA) (in PBS) as negative controls. Contact times of 120 s and disassociation times of 600 s were chosen and 12 mM HCl was used to regenerate before each new injection cycle.

### 2.6 Bioactivity assay

To assess the biological activity of IL15Rα fusions, their capability to stimulate CTLL2 cell proliferation in complex with IL15 was assessed.

In 96-well plates 50’000 cells were seeded per well in culture medium supplemented with different concentrations of F8IL15Rα, muIL15 peprotech, or the individual IL15Rα fusions in complex with equimolar amounts of muIL15 peprotech.

After incubation for 48 h in an incubator with 5% CO_2_ atmosphere at 37°C the cell proliferation was assessed with Cell Titer 96 Aqueous One Solution (Promega, Switzerland).

## 3 Results

### 3.1 Protein characterization

**Fig 1:**
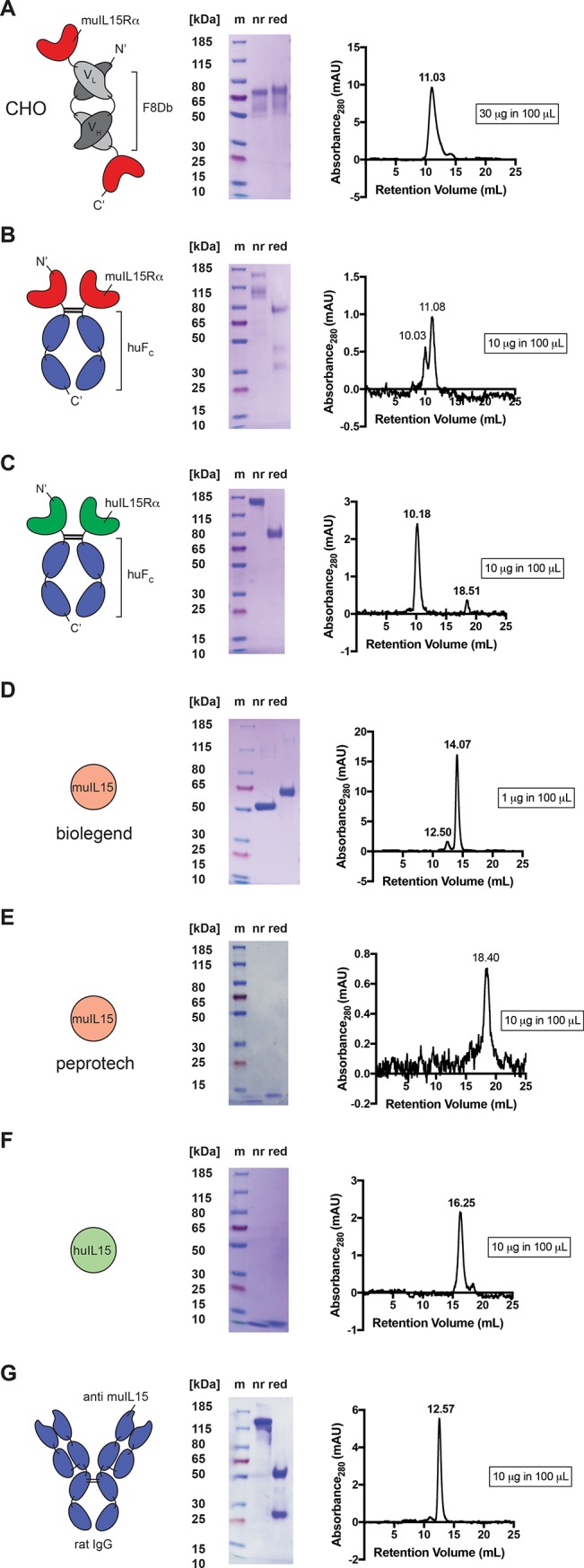
*In vitro* characterisation of produced and purchased proteins. Cartoon, SDS-PAGE gel and SEC profile of (A) the noncovalent dimer F8IL15Rα in diabody format (predicted monomer molecular mass 44.4 kDa), (B) muIL15RαFc (predicted monomer molecular mass 44.9 kDa, manufacturer information: 80–90 kDa, 42 kDa and 35 kDa in reducing conditions), (C) huIL15RαFc (predicted monomer molecular mass 45 kDa, manufacturer information: 75–90 kDa in reducing conditions), (D) muIL15 biolegend (13.3 kDa), (E) muIL15 peprotech (13.3 kDa), (F) huIL15 (12.9 kDa) and (G) aIL15mAb. CHO = Chinese hamster ovary, mu = mouse, F8Dd = F8 diabody, hu = human, Fc = crystallisable fragment, m = marker, IgG = immunoglobulin G, nr = nonreducing conditions and red = reducing conditions.

F8IL15Rα was cloned, produced transiently in CHO-S cells and purified to homogeneity, showing smeared bands in SDS-PAGE due to protein glycosylation (Fig 1A). Purchased muIL15RαFc, huIL15RαFc, muIL15 biolegend, muIL15 peprotech, huIL15 and aIL15mAb were also analysed by SEC and SDS-PAGE (Fig 1B–1G). In SDS-PAGE all IL15Rα fusions showed bands above their predicted molar mass, in line with the manufacturer’s specifications and with the glycosylated nature of the protein. Surprisingly, muIL15 biolegend exhibited an apparent molecular weight in SDS-PAGE of 50–60 kDa, while the predicted molar mass is 13kDa. Furthermore, the protein quantity was substantially greater than the nominal concentration provided by the manufacturer. This batch of protein was used for all further experiments, however, an additional batch of IL15 (muIL15 biolegend batch 2) was requested from the manufacturer solely for protein characterisation purposes and upon testing on SDS-PAGE gel showed main bands at 13 kDa (S1 Fig).

### 3.2 IL15 complexation experiments with SEC

**Fig 2:**
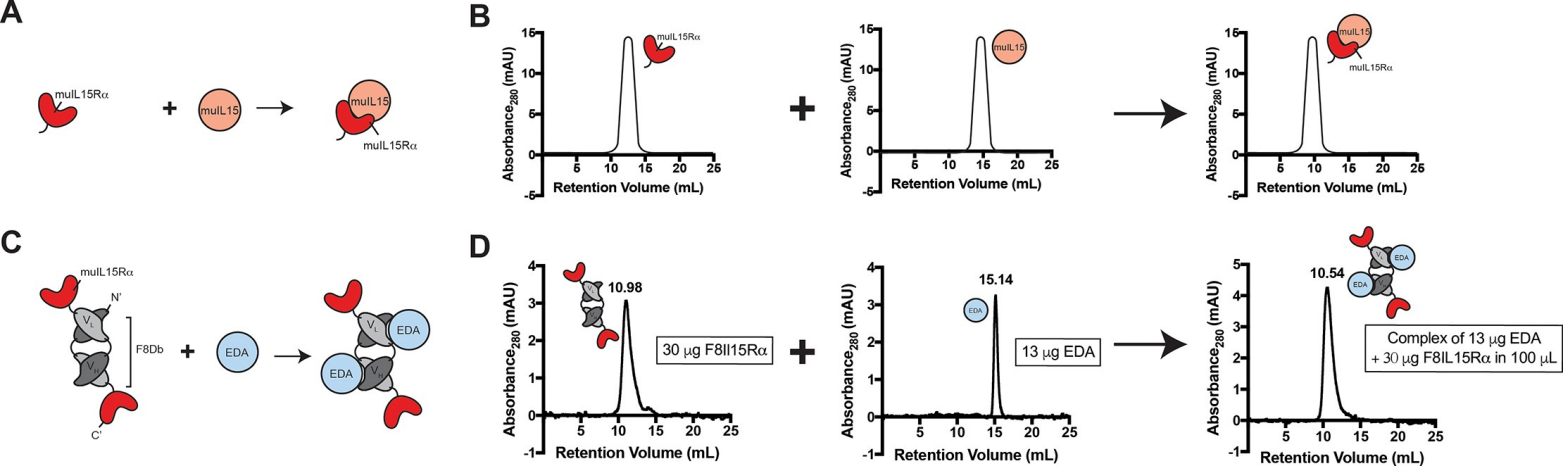
SEC complexation experimental scheme and positive control. (A+B) Scheme of the SEC complexation experiments visualizing (A) IL15Rα binding to IL15 by showing (B) the merging of the separate protein peaks into one single complex peak. (C) Positive control experimental scheme of F8IL15Rα binding to its cognate antigen EDA and (D) experimental data showing peak merging at equimolar amounts of individual complex components. EDA = extra-domain A of fibronectin, F8Ab = F8 antibody.

The ability of F8IL15Rα, muIL15RαFc and aIL15mAb to bind muIL15 and of huIL15RαFc to bind huIL15 was analysed on SEC. The binding of proteins can be visualized on SEC as separate peaks of the individual components merge into one single peak when the components are bound to each other (Fig 2).

**Fig 3:**
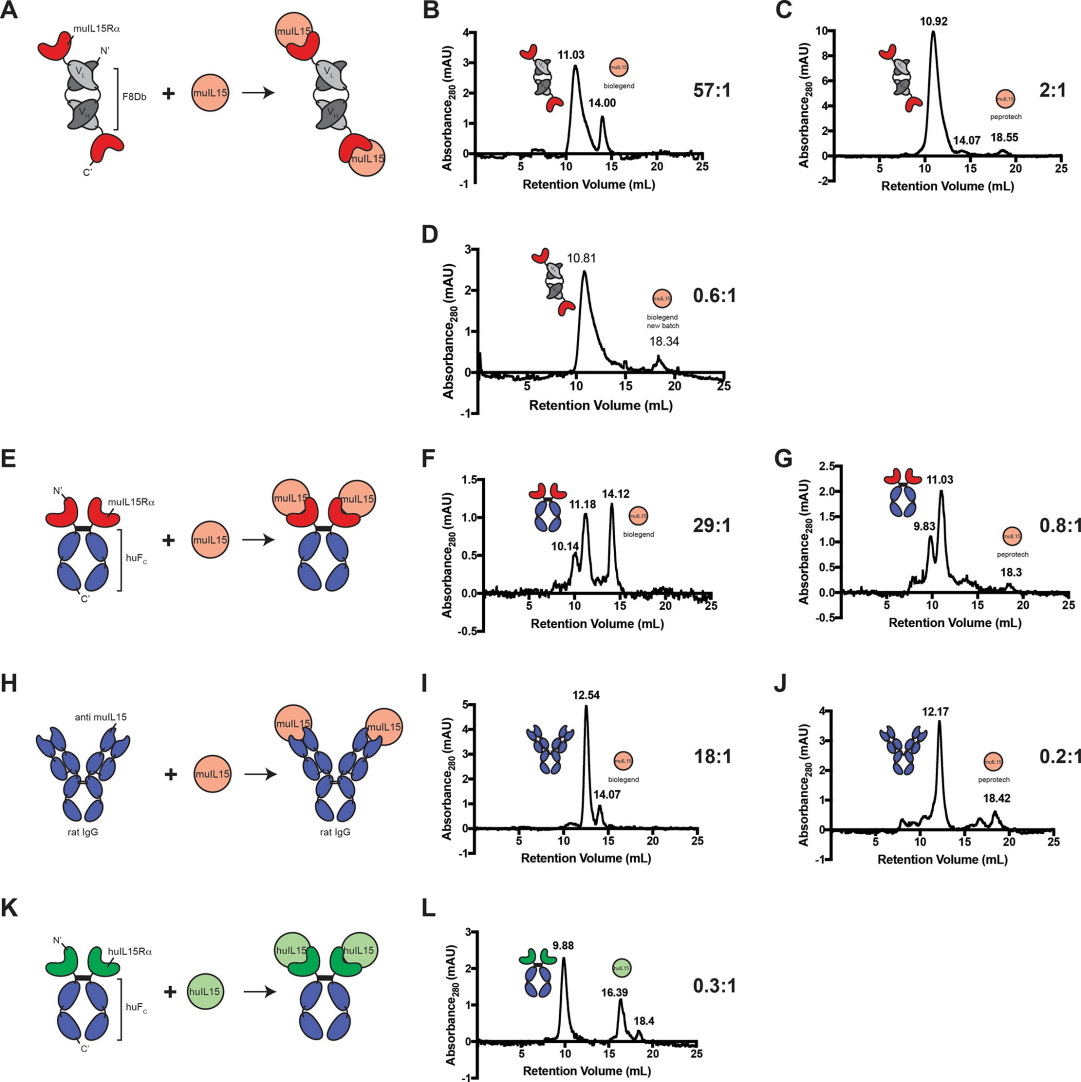
SEC complexation experiments. Cartoons and SEC profiles of the various IL15 binding proteins and IL15. The individual protein peaks (marked with cartoons) always remained visible and were not combined into a single complex peak. (A) F8IL15Rα binding to muIL15. (B) SEC profile using a F8IL15Rα:muIL15 biolegend ratio of 57:1. (C) SEC profile using a F8IL15Rα:muIL15 peprotech ratio of 2:1. (D) SEC profile using a F8IL15Rα:muIL15 biolegend batch 2 ratio of 0.6:1. (E) muIL15RαFc binding to muIL15. (F) SEC profile using a muIL15RαFc:muIL15 biolegend ratio of 29:1. (G) SEC profile using a muIL15RαFc:muIL15 peprotech ratio of 0.8:1. (H) aIL15mAb binding to muIL15. (I) SEC profile using a aIL15mAb:muIL15 biolegend ratio of 18:1. (J) SEC profile using a aIl15mAb:muIL15 peprotech ratio of 0.2:1. (K) huIL15RαFc binding to huIL15. (L) SEC profile using a huIL15RαFc:huIL15 ratio of 0.3:1.

None of the fusion proteins showed complex formation on SEC (Fig 3**)**. The individual components of the different complexes showed similar peak intensities to their characterization SEC profiles. Changing complexation conditions or the molar concentrations ratios did not show any effect (S2 Fig).

### 3.3 IL15 complexation experiments with SPR

**Fig 4:**
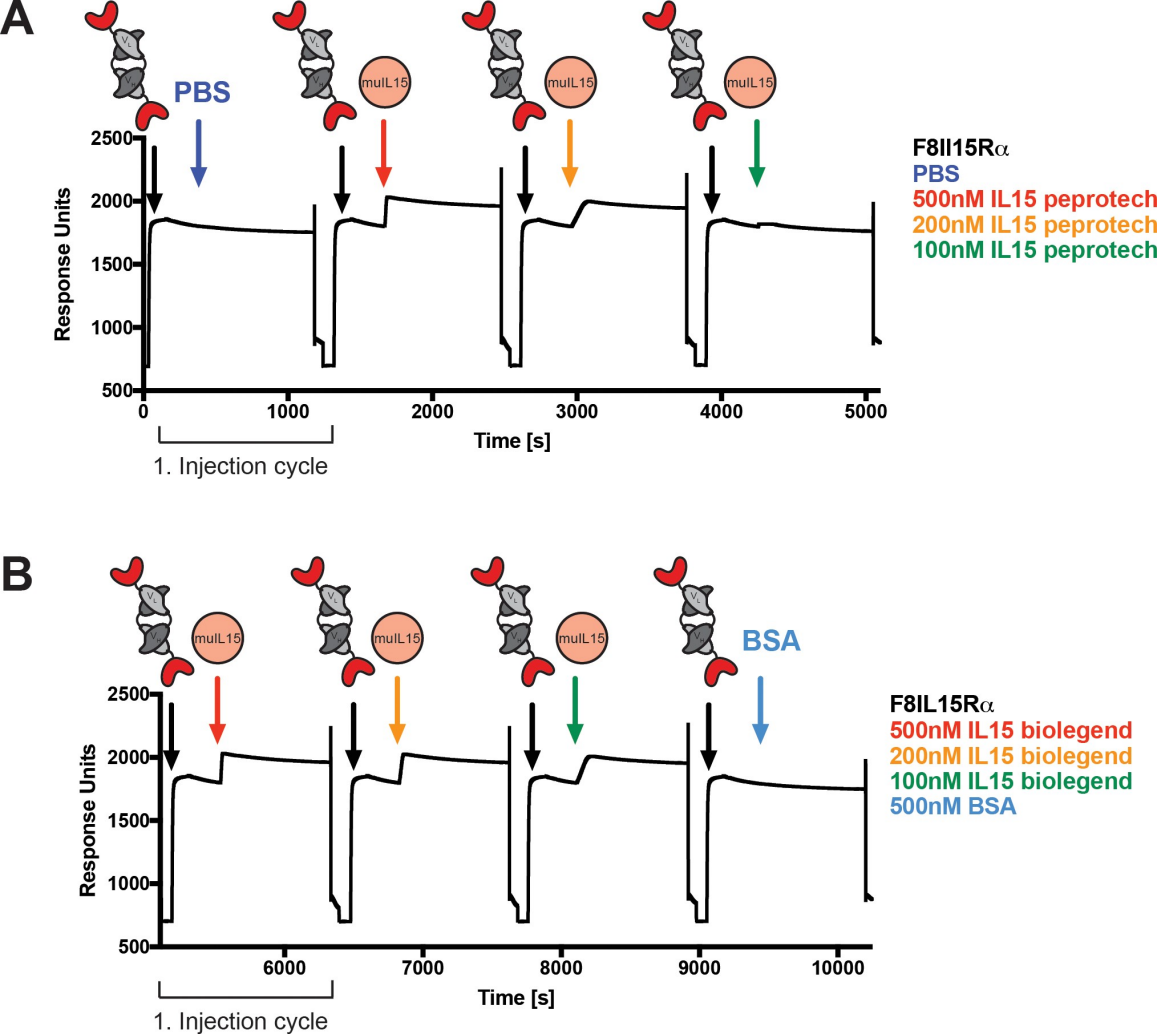
SPR binding experiment. In repeated cycles, F8IL15Rα was flowed over and bound to an EDA-coated chip followed by injection of a sample and subsequent regeneration to return to baseline. Timing of F8IL15Rα injections are marked with black arrows and cartoons, and timing of IL15, phosphate buffered saline (PBS) and bovine serum albumin (BSA) sample and control injections are marked by colourful arrows and cartoons. The corresponding sample names and concentrations are indicated in the same colour as the arrow. (A) Experiment using muIL15 peprotech as samples and PBS as a negative control. (B) Experiment using muIL15 biolegend as samples and BSA as a negative control.

The ability of F8IL15Rα to bind muIL15 biolegend and muIL15 peprotech was analysed using an EDA-coated chip with SPR (for SPR binding scheme, see S3 Fig). Binding of F8IL15Rα to EDA is evidenced in each injection cycle by the first signal increase from baseline (Fig 4). The second signal increase was observed as soon as the different IL15 concentrations were injected, indicating binding of IL15 to F8IL15Rα (Fig 4). When BSA or PBS were injected as negative controls, no second signal increase was observed. During regeneration between the separate injection cycles, the response units always returned back to baseline.

### 3.4 IL15Rα biological activity

**Fig 5:**
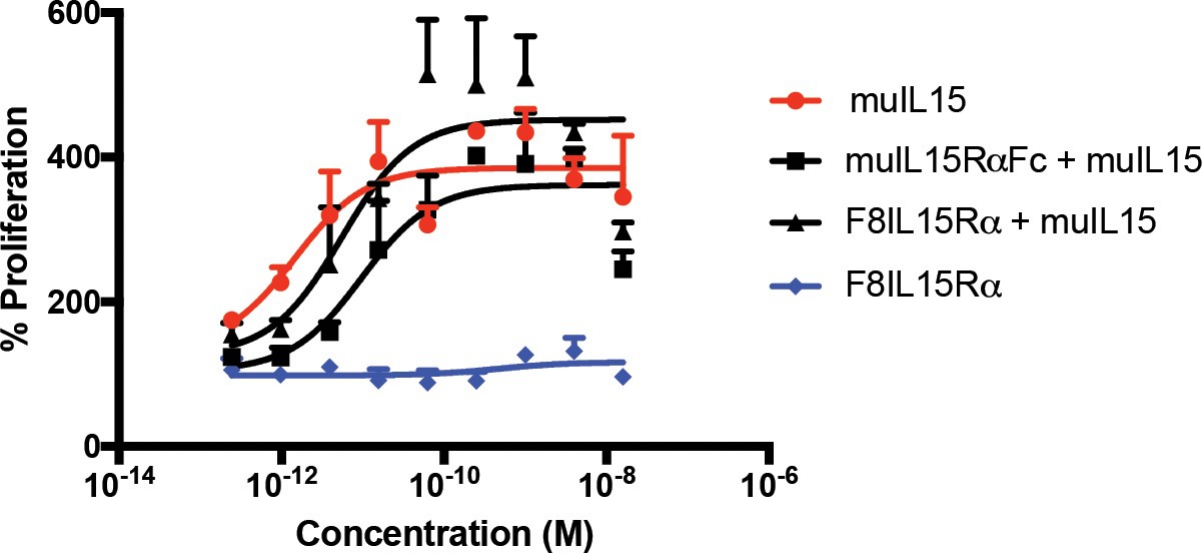
Biological activity of IL15Rα fusions. The biological activity of F8IL15Rα and muIL15RαFc complexed with equimolar amounts of muIL15 peprotech was assessed by their ability to stimulate the proliferation of CTLL2 cells (50’000cells/well) in triplicates. MuIL15 peprotech was used as a positive control and F8IL15Rα was used as a negative control.

As expected, IL15 preparations were able to increase cellular proliferation of CTLL2 cells. However, their activity was not potentiated by complexation with any of the IL15Rα fusion proteins (Fig 5), a feature that would have been expected based on previous reports on the potentiation of IL15 activity with the alpha subunit of its cognate receptor.[9] F8IL15Rα alone showed no activity and was used as a negative control.

## 4 Discussion

The F8IL15Rα fusion protein, consisting of the clinical-stage F8 antibody and the alpha subunit of the murine IL15 receptor, was cloned, expressed and purified to homogeneity. The protein displayed a high binding affinity towards both the cognate antigen (the alternatively-spliced EDA domain of fibronectin) and commercial preparations of murine IL15 in SPR analyses, performed on a BIAcore instrument. While F8IL15Rα could quantitatively titrate the cognate EDA antigen in SEC (Fig 2), no IL15-complex formation was visible on SEC and no potentiation of IL15 activity could be observed.

It is unclear as to why IL15 complex formation cannot be visualized on SEC, especially when F8IL15Rα binding to EDA is clearly visible. A validated and commercially available monoclonal antibody directed against IL15 was also unable to form a stable complex with the cognate antigen in SEC analysis. It is hypothetically possible that the gel filtration matrix may promote a dissociation of the complex between IL15 and its cognate receptor, but this feature would not be expected, considering the fact that the dissociation constant (Kd) and the kinetic dissociation constant (koff) for the receptor complex have been published to be approx. 38 pM and 1.4*10^−5^ s-1, respectively.[23]

It is surprising that no potentiation of IL15 biological activity way observed for F8IL15Rα or the bought IL15Rα fusion proteins, considering that muIL15RαFc, purchased from the same vendor, has been reported to increase CTLL2 cell proliferation in complex with muIL15. [9] There are numerous publications on the increase in biological activity of IL15 when complexed with its cognate receptor alpha chain.[3, 5, 9–12] However, the stable formation of complexes is not usually analysed apart from checking for increased IL15 activity. Further differences include other publications’ use of a molar excess of the receptor fusion proteins compared to IL15 rather than equimolar amounts as well as different methods of cellular proliferation assessment, such as [^3^H]-thymidine incorporation.[9]

ALT-803 is a soluble non-covalent IL15-complex currently in phase I and phase II clinical trials.[13] It consists of a mutated version of human IL15, in which the asparagine at position 72 was substituted by an aspartic acid to increase biological activity, and a human IL15Rα sushi-domain Fc fusion protein.[5] By complexing the mutated IL15 with the receptor fusion protein, IL15 biological activity and half-life are increased and extended.[5] ALT-803 is being suggested for use as a complementary immunomodulator for the treatment of solid as well as hematologic malignancies.[24] Interestingly, data on the intravenous application of ALT-803 in patients show a product half-life in serum of about two hours, which is very fast compared to the half-life of approved and established therapeutic Fc-fusions like the anti-rheumatic drug abatacept.[24, 25]

Another IL15 receptor based product being investigated in a phase I clinical trial for the treatment of metastatic and advanced solid tumours is hetIL-15, a fusion protein of IL15 directly linked to the human IL15Rα sushi-domain.[26] Furthermore, Genentech paid 120 Mio US$ for the rights to the Xencor IL15 pipeline, including their lead compound XmAb24306, a fusion of the IL15-IL15Rα complex to a bispecific Fc domain.[27, 28]

In view of the importance of IL15 in immunology research and for pharmaceutical applications, a better understanding of the binding properties of this cytokine with the cognate receptors would be highly desirable. It was strange to us that commercial and laboratory preparations of these products would not be able to form stable complexes in gel filtration experiments. We hope that this Communication may facilitate a better understanding of IL15 biology and that in the future the disconnect between BIAcore results (broadly used in the literature without orthogonal analytical investigations) and complex formation studies by SEC could be elucidated and overcome.

## Supporting information

S1 Fig *In vitro* characterisation of the new IL15 biolegend batch. (A) Cartoon, (B) SDS-PAGE gel and (C) SEC profile of the new batch of muIL15 biolegend (13.3 kDa) analysed after contact with the manufacturer.
(TIF)

S2 Fig Varying conditions of SEC complexation experiments with F8IL15Rα and muIL15 biolegend. SEC profiles of complexation experiments using F8IL15Rα and muIL15. (A–C) At fixed standard complexation conditions the molar concentration ratios of the proteins were varied. (D–F) At a fixed F8IL15Rα:muIL15 biolegend ratio of 57:1 the reaction conditions were varied. The individual protein peaks (marked with cartoons) remained visible and were not combined into a single complex peak. (A) SEC profile using a F8IL15Rα:muIL15 biolegend ratio of 114:1. (B) SEC profile using a F8IL15Rα:muIL15 biolegend ratio of 57:1. (C) SEC profile using a F8IL15Rα:muIL15 biolegend ratio of 1:1. (D) SEC profile where complexes were formed at 4°C for 20 h. (E) SEC profile where complexes were formed at room temperature (RT) for 15 h. (F) SEC profile where complexes were formed at 37°C for 0.5 h.
(TIF)

S3 Fig SPR binding scheme. Binding of F8IL15Rα to the chip and muIL15 to its cognate receptor increase the response units measured on SPR as visualized (A) in the cartoon and (B) graphically. (A+B) F8IL15Rα is flowed onto an EDA-coated chip where the F8 portion binds EDA, generating signal increase 1. Then IL15 is flowed onto the chip with F8IL15Rα still bound and IL15 binding to the IL15Rα portion generates signal increase 2. Through washing with an acidic solution the signal returns to baseline as the complex disassociates.
(TIF)
