# Supplementary figures and images for: Design and characterisation of a novel interleukin-15 receptor alpha fusion protein and analysis of interleukin-15 complexation
Source: PLoS One. 2019 Jul 26;14(7):e0219313. doi: 10.1371/journal.pone.0219313 (PMC6660064; doi:10.1371/journal.pone.0219313)

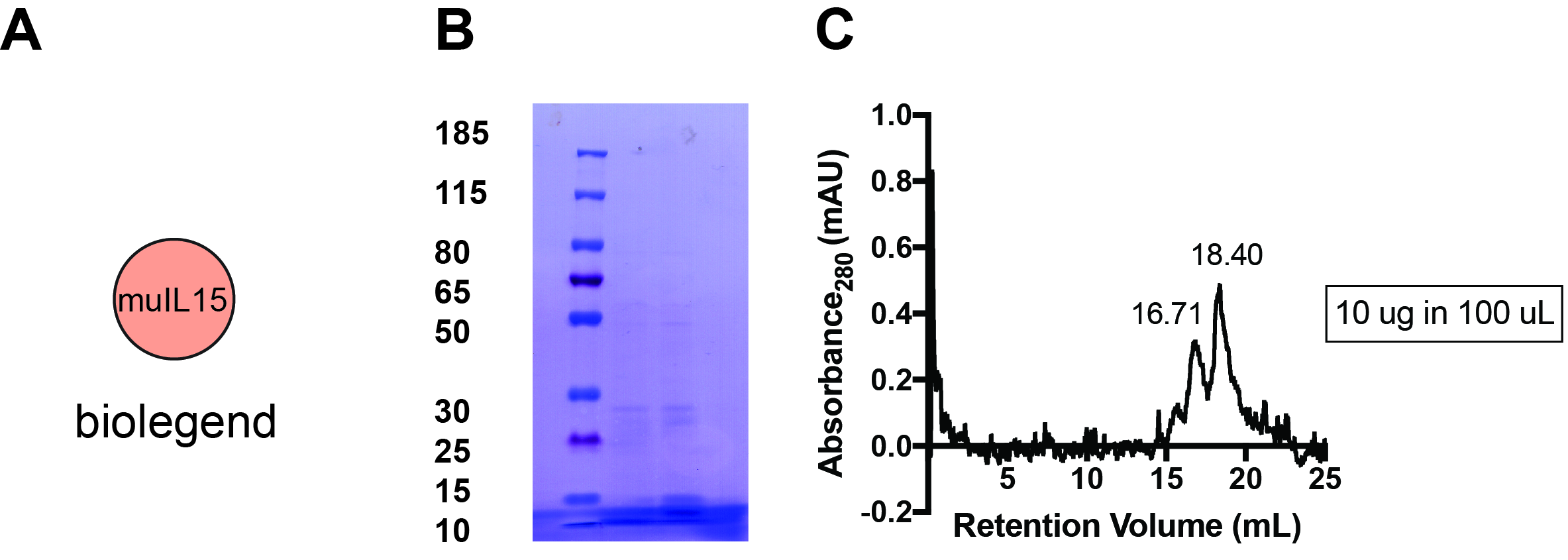

Supplement: S1 Fig — (A) Cartoon, (B) SDS-PAGE gel and (C) SEC profile of the new batch of muIL15 biolegend (13.3 kDa) analysed after contact with the manufacturer. (TIF) [file pone.0219313.s001.tif]

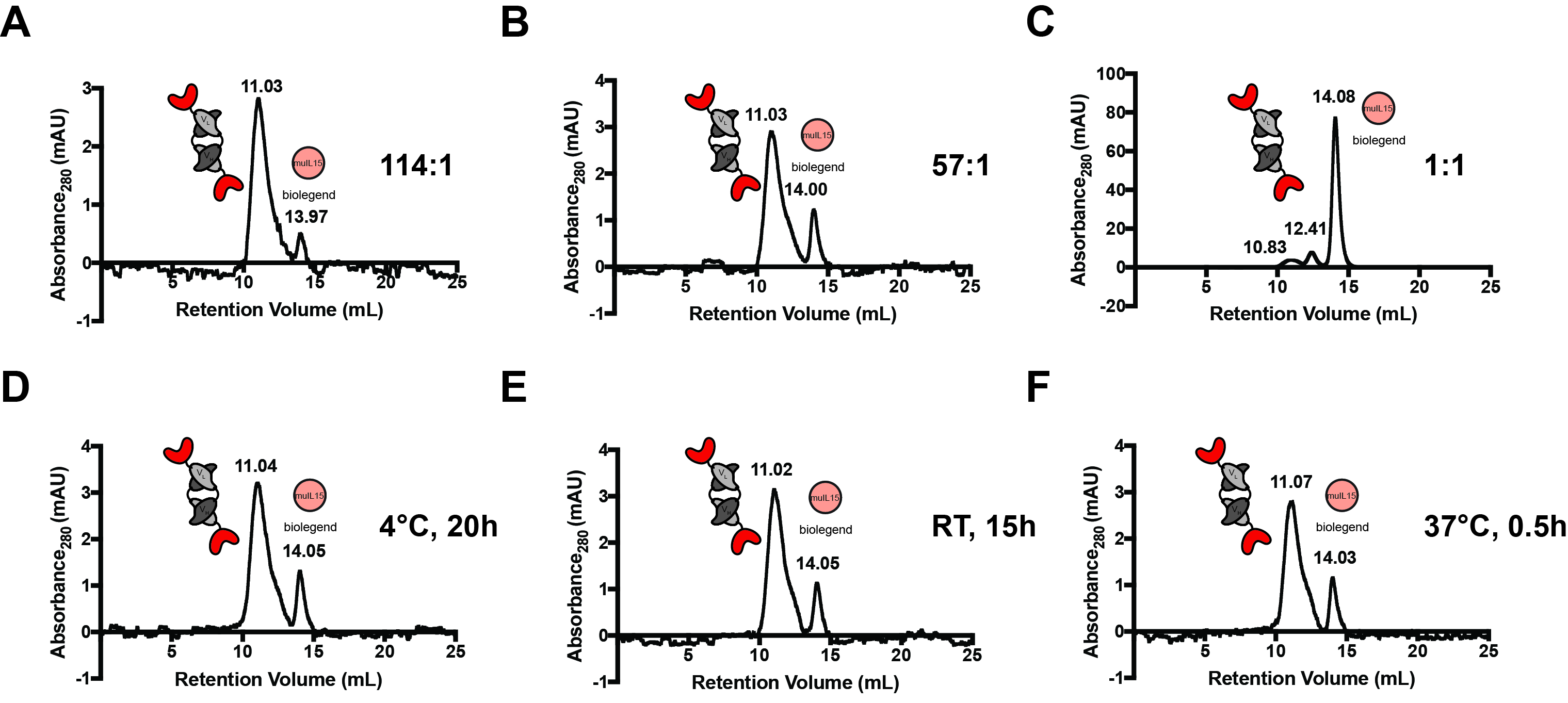

Supplement: S2 Fig — SEC profiles of complexation experiments using F8IL15Rα and muIL15. (A–C) At fixed standard complexation conditions the molar concentration ratios of the proteins were varied. (D–F) At a fixed F8IL15Rα:muIL15 biolegend ratio of 57:1 the reaction conditions were varied. The individual protein peaks (marked with cartoons) remained visible and were not combined into a single complex peak. (A) SEC profile using a F8IL15Rα:muIL15 biolegend ratio of 114:1. (B) SEC profile using a F8IL15Rα:muIL15 biolegend ratio of 57:1. (C) SEC profile using a F8IL15Rα:muIL15 biolegend ratio of 1:1. (D) SEC profile where complexes were formed at 4°C for 20 h. (E) SEC profile where complexes were formed at room temperature (RT) for 15 h. (F) SEC profile where complexes were formed at 37°C for 0.5 h. (TIF) [file pone.0219313.s002.tif]

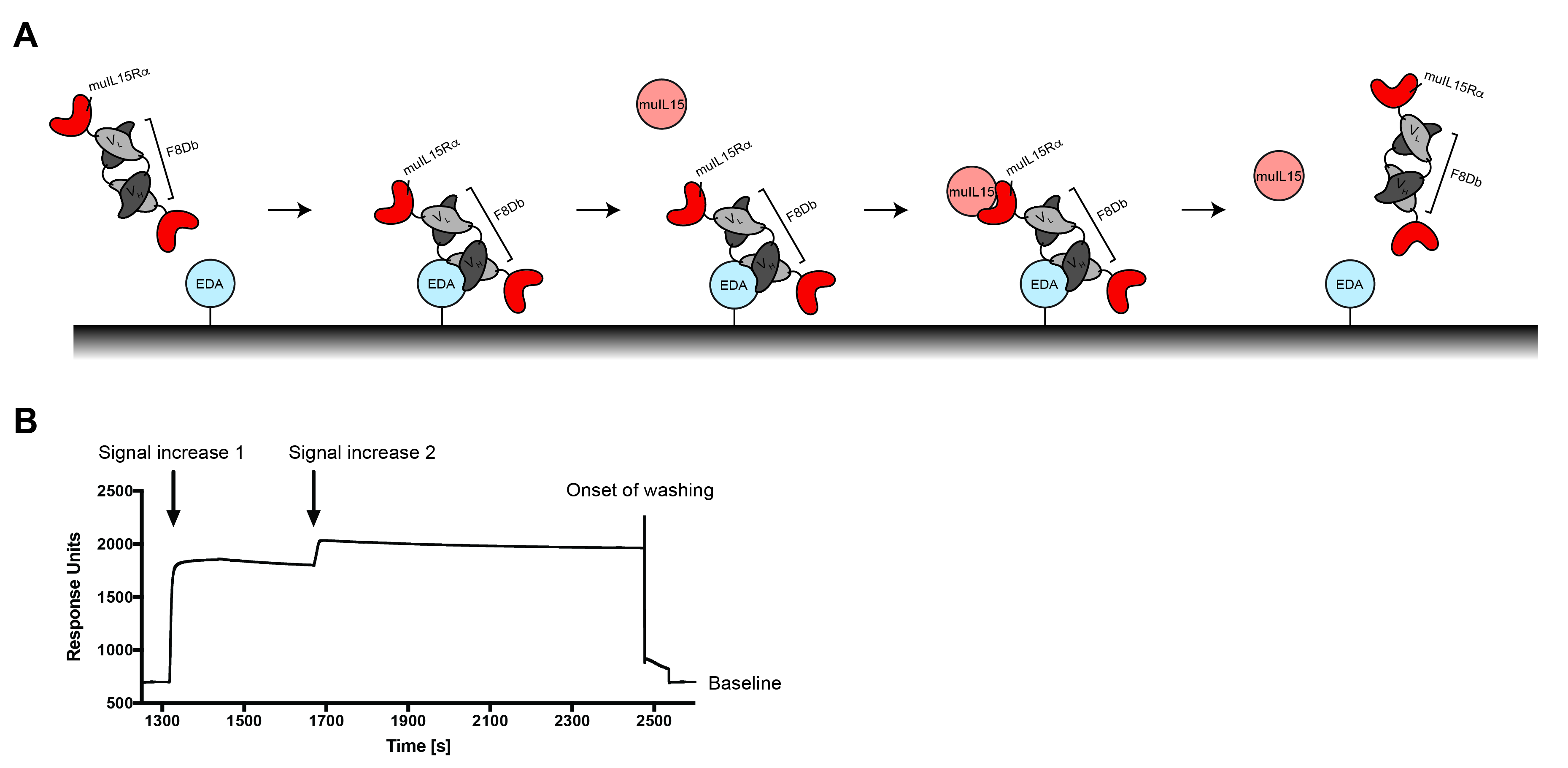

Supplement: S3 Fig — Binding of F8IL15Rα to the chip and muIL15 to its cognate receptor increase the response units measured on SPR as visualized (A) in the cartoon and (B) graphically. (A+B) F8IL15Rα is flowed onto an EDA-coated chip where the F8 portion binds EDA, generating signal increase 1. Then IL15 is flowed onto the chip with F8IL15Rα still bound and IL15 binding to the IL15Rα portion generates signal increase 2. Through washing with an acidic solution the signal returns to baseline as the complex disassociates. (TIF) [file pone.0219313.s003.tif]
